# Insights into SARS-CoV-2: Small-Molecule Hybrids for COVID-19 Treatment

**DOI:** 10.3390/molecules29225403

**Published:** 2024-11-15

**Authors:** Maria Luisa Navacchia, Caterina Cinti, Elena Marchesi, Daniela Perrone

**Affiliations:** 1 Institute for Organic Synthesis and Photoreactivity (ISOF), National Research Council of Italy (CNR), 40129 Bologna, Italy; caterina.cinti@cnr.it; 2 Department of Chemical, Pharmaceutical and Agricultural Sciences, University of Ferrara, 44121 Ferrara, Italy; mrclne@unife.it; 3 Department of Environmental and Prevention Sciences, University of Ferrara, 44121 Ferrara, Italy

**Keywords:** molecular hybridization, small molecules, natural product hybrids, antiviral activity, drug design, COVID-19, SARS-CoV-2 M^pro^

## Abstract

The advantages of a treatment modality that combines two or more therapeutic agents with different mechanisms of action encourage the study of hybrid functional compounds for pharmacological applications. Molecular hybridization, resulting from a covalent combination of two or more pharmacophore units, has emerged as a promising approach to overcome several issues and has also been explored for the design of new drugs for COVID-19 treatment. In this review, we presented an overview of small-molecule hybrids from both natural products and synthetic sources reported in the literature to date with potential antiviral *anti*-SARS-CoV-2 activity.

## 1. Introduction

Coronavirus disease (COVID-19), caused by severe acute respiratory syndrome coronavirus 2 (SARS-CoV-2), is an infectious disease that is mainly responsible for acute respiratory symptoms but is also associated with other symptoms such as hypertension, thrombosis (blood coagulation in the vessels), pulmonary embolism, heart attack and stroke. A sanitary emergency, generated by the pandemic of COVID-19 on the global population causing over 7 million deaths to date [1], has dramatically impacted public healthcare systems and has posed a huge challenge to global health for searching strategies mitigating the damage caused by the disease. Several strategies have been carried out to fight the pandemic. Unprecedented vaccine development and global mass vaccination represent a very successful approach; however, a relevant proportion of the global population remains unvaccinated due to the inability to vaccinate owing to preexisting conditions or recalcitrance due to personal beliefs. Moreover, the appearance of new SARS-CoV-2 variants reduces the effectiveness of existing vaccines that are not efficient in preventing disease onset [2]. Among the different therapeutic strategies proposed to counteract SARS-CoV-2 infection, the development of small-molecule antiviral drugs targeting viral proteins required for virus replication can help to overcome SARS-CoV-2 immune escape since the mechanism of action is unaffected by spike protein changes. In this light, several direct-acting antiviral small-molecule drugs approved for other therapeutic applications have been repurposed [3]. Among them, remdesivir [4] and molnupiravir [5], nucleoside analogs targeting viral genome replication, as well as paxlovid [6,7,8], which combines nirmatrelvir, an inhibitor of the viral protease chymotrypsin-like cysteine (3CLpro), with the main protease (M^pro^) inhibitor ritonavir, were quickly developed and approved for human use. However, these antiviral drugs present significant limitations in clinical use, such as the exclusive intravenous administration of remdesivir, the outcome that molnupiravir is not effective in reducing mortality and hospitalization even though it has been recognized to accelerate the rehabilitation of COVID-19 patients [9,10], and drug–drug interactions with concomitant medications that limit the use of paxlovid [11]. Therefore, the research for complementary antiviral agents, which still represent an important therapeutic treatment option, remains a pressing matter [12].

**Figure 1:**
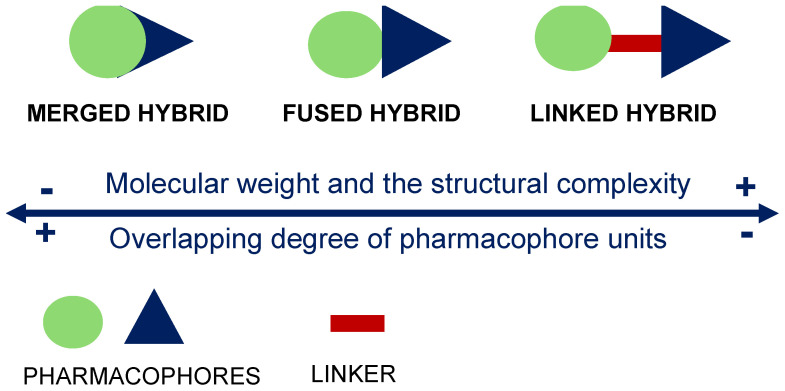
Design strategy for hybrid compounds.

In this scenario, we reported herein advances in research on small hybrid molecules potential antiviral candidates against SARS-CoV-2 with regard to the in silico and in vitro data reported in literature to date. Molecular hybridization (MH) is a well-established strategy in drug discovery for developing multitarget drug candidates for complex diseases. MH is a useful tool to optimize the therapeutic effect of active products by improving bioavailability and reducing toxicity as well overcoming multidrug resistance. MH consists of the conjugation through covalent bonds of two or more pharmacophore units, resulting in a single molecule multiple targets with improved pharmacological and pharmacokinetic profiles respect to the parent pharmacophores used alone or in combination [13]. The hybridization of two active molecules can be achieved in different ways: merged and fused hybrids are obtained by using functional groups initially present on the combination partners, whereas the introduction of a linker unit, not present in either of the starting pharmacophores, leads to linked hybrids (Figure 1). 

It is worth noting that the linker itself can influence biological activity by improving the pharmacological, pharmacokinetic or physiochemical profiles of the resulting hybrid, as in the case of a triazole ring introduced by click chemistry, a cycloaddition reaction widely employed in the field of medicinal chemistry [14,15]. The generation of fused, merged or linked hybrids is generally driven by the nature of the targets, the availability of functional groups and the chemical feasibility. The MH approach leads to larger and more complex less drug-like molecules. When the overlap between the combination partners is maximized and, in turn, the size of the resulting hybrids is minimized, drug-like features may be retained. Thus, in principle, merged and fused hybrids might have more chances to keep drug-like properties compared to linked hybrids. From a synthetic point of view, many reactions can be employed for the conjugation. For instance, condensation reaction represents a simple and efficient method to achieve conjugates leading to ether, ester or amide derivatives depending on the nature of the reactive moieties available on the pharmacophores. The click chemistry, involving a terminal carbon–carbon triple bond and an azido group, is also widely employed to obtain linked hybrids through the formation of the biocompatible triazole ring stable under both chemical and enzymatic conditions [14,16].

MH can involve both natural and synthetic products [17,18]. In particular, the molecular complexity of biologically active natural products makes them ideal templates for the design of new hybrid drugs. In the case of the COVID-19 emergency, the repurposing of approved drugs as pharmacophores for MH was also taken into account to accelerate the discovery of new cures and possibly shorten the approval time. 

## 2. Coronaviruses Overview

Insight into coronaviruses and their mechanisms of action was reported to support a rational design on the basis of new hybrids as well as the rationale behind the choice of repurposed drugs with potential antiviral *anti*-SARS-CoV-2 activity.

### 2.1. Coronaviruses Genomic Organization 

Coronaviruses (CoVs) are known as harmless respiratory pathogens to humans. Currently, six human coronaviruses have been confirmed; two of them belong to the alpha-coronavirus genus (HCoV-NL63 and HCoV-229E) and four belong to the beta-coronavirus genus (HCoV-OC43, HCoV-HKU1, SARS-CoV, and MERS-CoV). All coronaviruses typically contain positive-strand RNAs that differ in size, ranging from 26 kb to 32 kb. The genome includes a variable number of open reading frames (ORF) from six to fourteen, in the case of SARS-CoV-2. The first one (ORF1) is the largest, distinguished in two main transcriptional units, ORF1a and ORF1ab, encoding two overlapping polyproteins, the pp1a and pp1ab, respectively. The pp1a and pp1ab polyproteins embed 11 and 16 non-structural proteins (nsp1-16), respectively, which form the complex replicase machinery. Of the 16 nsps, the main protease (M^pro^), also known as chymotrypsin-like cysteine protease (3CLpro), encoded by nsp5, and the papain-like protease (PL^pro^), encoded by nsp3, cleave the non-structural proteins into the two overlapping pp1a and pp1ab polyproteins. Both PL^pro^ and M^pro^, together with other non-structural proteins, Helicase (Hel) and RNA-dependent RNA polymerase (RdRp), are involved in the transcription and replication of CoVs and have shown high conserved genome sequences, sharing more than 90% sequence similarity with the corresponding beta-coronavirus genus [19,20]. Since PL^pro^ and M^pro^ are considered key enzymes in the viral life cycle, playing a fundamental role in viral gene expression and replication, they can be viewed as two attractive targets for anti-CoVs drug design. It is important to note that the substrate specificity of proteolytic enzyme M^pro^ is dissimilar to human proteases since it exclusively cleaves polypeptide sequences after a glutamine residue, making it an ideal drug target. Differentially, the protease PL^pro^ recognizes the C-terminal sequence of ubiquitin that is also present in the host cells. Therefore, substrate-derived inhibitors of PL^pro^ would be expected to also inhibit host–cell deubiquitinases, making drug discovery campaigns against PL^pro^ challenging [21,22,23].

**Figure 2:**
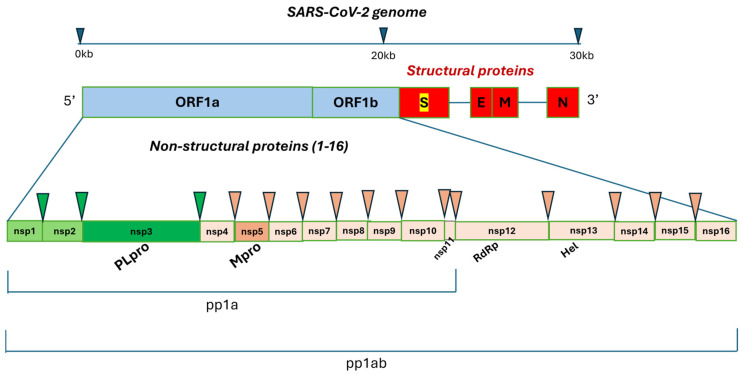
Schematic representation of the genome organization and functional domains for SARS-CoV-2. The single-stranded RNA genome of SARS-CoVs has two large genes, the ORF1a and ORF1b genes, which encode 16 non-structural proteins (nsp1–nsp16) that are highly conserved throughout coronaviruses. The structural genes encode the structural proteins, spike glycoprotein (S), envelope (E), membrane (M) and nucleocapsid (N), which are common features of all coronaviruses. Polyproteins pp1a and pp1ab embed 11 and 16 non-structural proteins (Nsps), respectively; the green and pink triangles indicate the cleavage sites of the protease PL^pro^ and M^pro^, respectively. Fifteen sites where polyproteins pp1a and pp1ab are cut by proteases are represented with arrows. PL^pro^ cleaves at three distinct sites while M^pro^ cleaves at twelve distinct sites, including those for RNA-dependent RNA polymerase (RdRp) encoded by nsp12 and Helicase (Hel) encoded by nsp13.

The remaining ORFs of coronaviruses encode accessory and structural proteins, including spike surface glycoprotein (S), small envelop protein (E), matrix protein (M) and nucleocapsid protein (N), which are essential for virus–cell-receptor binding and virion assembly for RNA-dependent RNA polymerase (RdRp) encoded by nsp12 and Helicase (Hel) encoded by nsp13 (Figure 2). 

### 2.2. Mechanism of Action of CoVs into Host Cells 

In humans, coronaviruses (CoVs) usually cause mild to moderate upper-respiratory tract illnesses; however, the rarer forms of CoVs belonging to Betacoronavirus have generated Middle East respiratory syndrome (MERS-CoV) and severe acute respiratory syndrome (SARS-CoV), which in some cases have been lethal. Both syndromes have predominantly respiratory manifestations, but extrapulmonary features may occur in severe cases such as thrombosis, renal failure, heart attack and stroke. 

Viruses infecting mammalian cells, activating specific pattern recognition receptors and immune signal transduction, result in pro-inflammatory cytokine production and the activation of innate immunity [24].

Viral infection stimulates the initiation of a complex series of events characterized by the early response of virus-infected cells to the innate production of cytokines and the induction of emergency innate immune response of neutrophils and macrophages. These events subsequently engage and amplify NK and T cells- mediated adaptive immune response for the production of further proinflammatory cytokines that are the basis of the cytokine storm observed in COVID-19 patients. The elevated circulating levels of cytokines impact a wide range of physiological processes and are associated with a variety of infections, having a direct role in the activation of *anti*-microbial effector functions and in providing regulatory signals for immune response [25]. The clinical consequence of elevated circulating levels of cytokines (most notably IL-1, Il-2, IL-6 and tumor necrosis factor TNF) is systemic inflammation that leads to progressive organ failure and cell death [26]. Apart from triggering inflammatory and immune responses, many viral infections can cause programmed cell death in infected cells. Evidence suggests that the induction of cell death by coronaviruses may significantly contribute to viral infection and pathogenicity [27].

Organ dysfunction and tissue damage have been observed as consequences of SARS-CoVs infection. It has been reported that SARS-CoV-2 coronavirus spike protein-induced apoptosis can be regulated in host cells via the increased reactive oxygen species (ROS), which inhibits the phosphoinositide 3-kinase (PI3K)/AKT/mammalian target of rapamycin (mTOR) pathways [28].

Although cell death is an effective host defense strategy, hyperactivation of the *anti*viral response and inflammatory cell death can cause systemic inflammation and pathology [27]. 

In the case of SARS-CoV and SARS-CoV-2, these viruses enter the host cells mainly by targeting the Angiotensin-Converting Enzyme 2 (ACE2) receptors, which are expressed in most mammalian cells, such as cardiac muscle cells, cardiac fibroblasts, the coronary vascular endothelium, kidneys, the liver, the small intestine, testes, the brain, lung alveolar epithelial cells, leukocytes, arterial cells and venous endothelial cells, as well as arterial smooth muscle cells [29,30]. 

**Figure 3:**
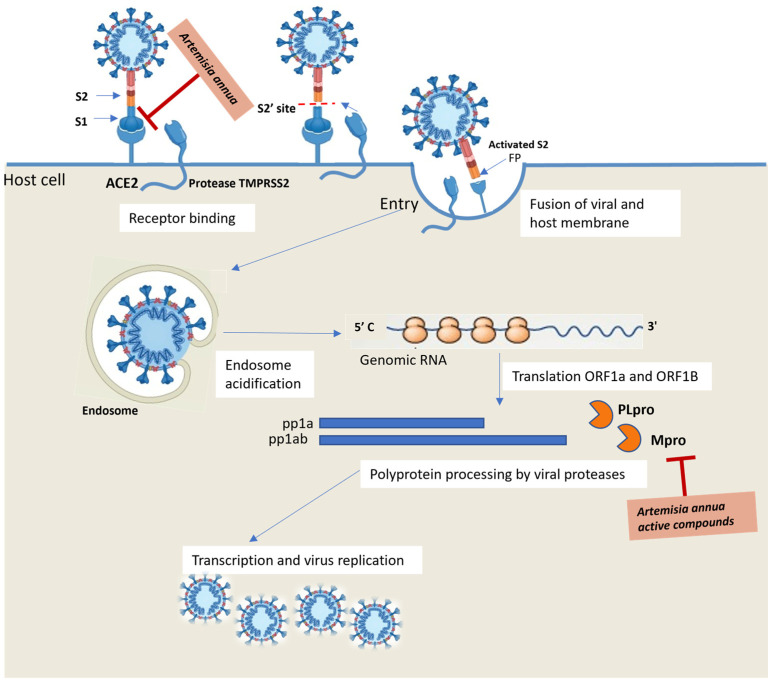
Schematic representation of virus infection and replication mechanism in host cell.

SARS-CoV and SARS-CoV-2 interact with ACE2 receptors via the surface glycoprotein S (homotrimer) [31]. The *N*-terminal of the S glycoprotein contains a peptide signal via the S1 and S2 subunits. The S1 subunit binds to the peptidase domain of ACE2 via the receptor binding domain (RBD) and the S2 subunit mediates the fusion of the viral membrane and host cell membrane and facilitates viral genomes’ entry into the host cells. Shang et al. reported that, unlike SARS-CoV, SARS-CoV-2 shows an increased affinity with ACE2 due to amino acid replacement in the RBD of protein S, hypothesizing that this is the reason for the high rate of the virus spread [32]. Once the virus binds to ACE2 receptors, the fusion peptide (FP) in the S2 subunit interacts with lipid layers in the host cell membrane and induces the fusion of the virus and host membranes and the formation of endosomes, in which cysteine proteases cathepsin B and L and transmembrane serine protease TMPRSS2 cleave protein S and facilitate the release of viral genomes into the cytoplasm [33]. In cells, virus amplification takes place by viral RNA polymerase, and the viruses then infect the surrounding cells (Figure 3). 

Although SARS-CoVs’ entry into host cells is mainly dependent on its interaction with ACE2 receptors, studies on patient samples have described a downregulation of the ACE2 expression in SARS-CoV-2-infected cells similar to SARS-CoV with implications for circulatory homeostasis [34]. 

Lu et al. explored the mechanism by which SARS-CoV-2 downregulates ACE2 in in vitro and in vivo models and suggest that the virus induces clathrin- and AP2-dependent endocytosis, leading to ACE2 degradation in the lysosome [35].

ACE2 viral-induced dysregulation changes the equilibrium of the ACE2-catalyzed reaction with the consequent accumulation of ACE2 substrates, such as Angiotensin II, apelin-13 and dynorphin-13, and decreases the concentration of products such as Angiotensin (1–7), Angiotensin (1–9), apelin-12 and dynorphin-12 in the human body. Substrate accumulation ultimately induces inflammation, angiogenesis, thrombosis and neuronal and tissue damage, while diminished products lead to the loss of *anti*-inflammatory, *anti*-thrombotic and *anti*-angiogenic responses.

Recent reports of excessive inflammation signaling, leading to elevated serum cytokine levels associated with coronavirus disease (COVID-19) have raised questions about the relationship between cytokine storms and severe pulmonary and cardiovascular complications associated with this infection [26]. In SARS-CoV-2 patients, the cytokine storms seem to be mainly due to Angiotensin II accumulation and concomitant reduction in the concentration of positive *anti*-inflammatory, *ant*i-thrombotic and *anti*-fibrotic functions Angiotensin (1–7) and Angiotensin (1-9) factors. In COVID-19 patients, either Angiotensin II accumulation or the reduced concentration of Angiotensin (1–7) and Angiotensin (1-9) are essentially due to dysregulation, internalization and shedding of ACE2 receptors. 

**Figure 4:**
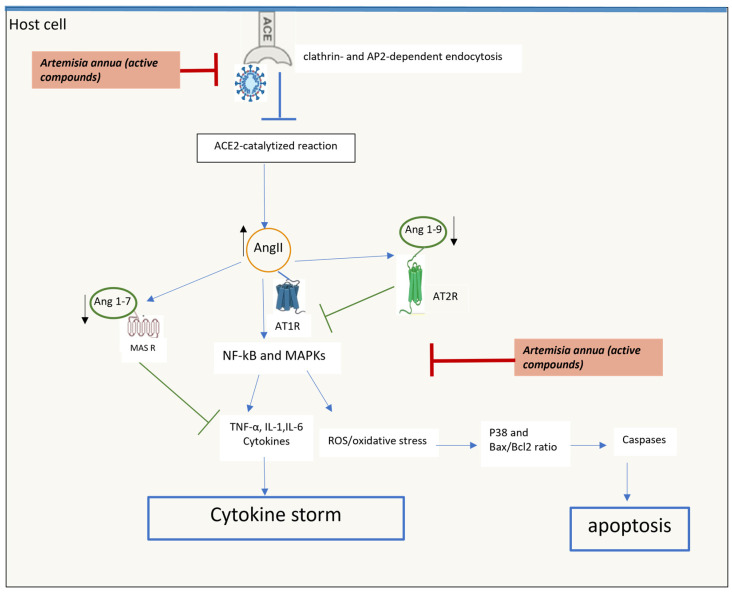
ACE2 viral-induced dysregulation and inflammatory signaling in host cell.

Angiotensin II stimulates inflammatory responses in leukocytes, endothelial cells and smooth muscle cells by activating NF-κB signaling, enhancing the transcription of TNF-α, IL-1 and Interleukin 6 (IL-6) inflammatory cytokines, as well as different chemokine and adhesive molecules such as VCAM-1 and ICAM-1. On the other hand, Angiotensin (1–7), throughout the binding of MAS receptors (MASR), can attenuate inflammatory responses by reducing neutrophil influx, downregulating CXC chemokine ligand (CXCL); IL-6, TNF-α and IL-1b cytokines; and Endothelin-1 (a vasoconstrictor and monocyte chemoattractant protein-1 (MCP-1)). Similar to Ang (1-7), Ang (1-9) are produced from AngII in a reaction catalyzed by ACE2 receptors. Ang (1-9), throughout the binding to AT2R, inhibit the AngII–AT1R signaling axis and balance the vasoconstrictive to vasodilatory axis in the heart, thus improving cardiovascular conditions. Ang (1-9)/AT2R-derived signaling also reduces inflammation and tissue fibrosis mainly in the heart and lungs, downregulating proinflammatory cytokines such as IL-6, IL1b, TNF-α and MCP-1 [36,37] (Figure 4). 

## 3. Design of Hybrid Molecules

Considering that SARS-CoV-2 has disseminated globally and is likely to continue circulating in humans with the possible emergence of new variants that may render vaccines less effective, the discovery and development of new virus-based and host-based therapeutic options are urgently needed. Antivirals targeting conserved viral components, such as spike proteins and proteases, or host targets and new therapeutics that can precisely modulate the immune response during infection could be possible approaches to reduce the harmful effect of viral infection. 

### 3.1. Artemisinin-Based Hybrids 

Several antimalarial drugs have been repurposed to tackle the COVID-19 pandemic [38], including artemisinin, a highly effective bioactive component of *Artemisia annua* (*A. annua*) [39]. Active components of *A. annua* have been reported to have effective antiviral properties and immunosuppressive effects in vivo. In particular, artemisinin is reported to decrease the infiltration of immunomodulatory cells and inflammatory cytokines [40]. Belonging to the active component of *A. annua*, isorhamnetin [41] and quercetin can prevent the SARS-CoV-2 virus from entering by disrupting the viral S protein Angiotensin-converting enzyme 2 (ACE2) receptor interface and can also interfere with virus replication by interacting with SARS-CoV protease M^pro^ (3CLpro) [42]. 

Recently, a network pharmacology strategy has been used to predict the main active compounds and key targets of *A. annua* for the treatment of COVID-19 [43]. From this analysis, Tang et al. postulated that *A. annua* acts on COVID-19 mainly by regulating inflammatory responses, transcription and proliferation. In particular, molecular docking simulation and computing binding affinity identified artemisinin, quercetin, isorhamnetin and kaempferol as the main potentially active compounds of *A. annua* to fight virus infection. An excellent binding affinity of these compounds with 7 key target inflammatory mediators related to the pathogenesis of COVID-19 have been identified and referred to vascular endothelial growth factor-A (VEGF), the proinflammatory TNF cytokine, MAPKs and pro-apoptotic caspases and p53 transcription factor (Figure 4). An excellent binding affinity of these compounds with 7 key target inflammatory mediators related to the pathogenesis of COVID-19 have been identified and referred to vascular endothelial growth factor-A (VEGF), the proinflammatory TNF cytokine, MAPKs and pro-apoptotic caspases and p53 transcription factor (Figure 4).

**Figure 5:**
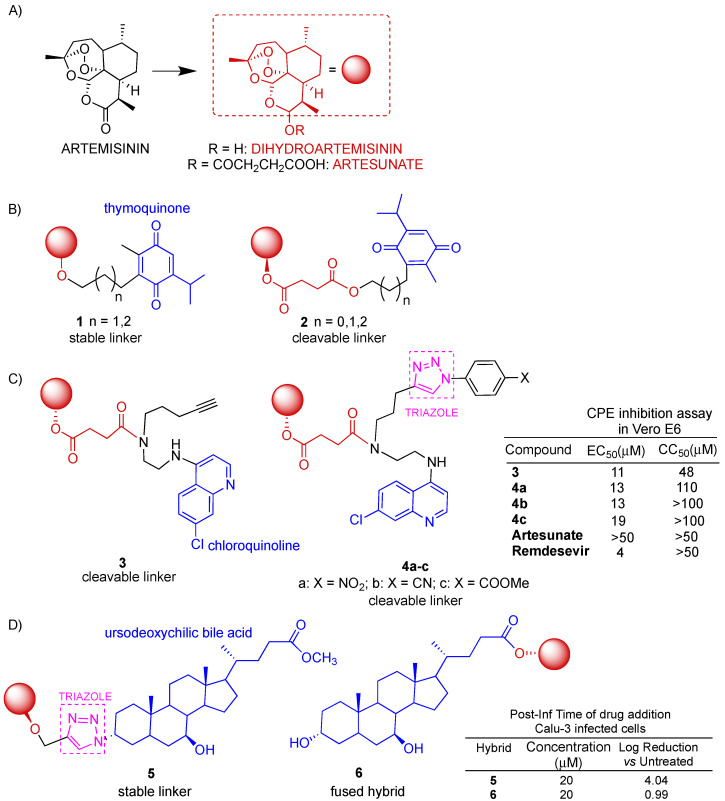
Artemisinin-derived hybrids and selected biological data. (**A**): molecular structures of artemisinins; (**B**): dihydroatemisinin–thymoquinone hybrids; (**C**): molecular structures of dihydroatemisinin–quinoline hybrids; (**D**): molecular structures of dihydroatemisinin–ursodeoxycholic bile acid hybrids.

Among artemisinin and its derivatives, collectively called artemisinins, dihydroartemisinin (Figure 5A) was found to be able to inhibit SARS-CoV-2 replication in vitro by decreasing viral protein production [44]. However, the clinical application of artemisinins is limited by their short half-life, poor solubility and lack of bioavailability. In order to overcome the unfavorable properties of artemisinins above-reported, MH has been widely employed over recent decades to design artemisinin-derived hybrids for cancer therapy [17]. Dihydroartemisinin *anti*-SARS-CoV-2 activity, together with suitable chemical features for conjugation, has encouraged MH with the aim to obtain new hybrid molecules that can act synergistically and improve upon the simple drug combination. 

Thymoquinone (Figure 5B) is a monoterpene obtained from *Nigella sativa*’s black seed oil characterized by wide-ranging pharmacological properties, including antioxidant, *anti*-inflammatory, antidiabetic, antihistaminic, antimicrobial, anticonvulsant and anticancer effects [45]. The pandemic also prompted the exploration of thymoquinone antiviral potential against SARS-CoV-2. Recent studies reported that thymoquinone has a high potential of binding at the SARS-CoV-2/ACE2 interface; therefore, it could be predicted to be a plausible inhibitor to disrupt viral–host interactions [46]. Moreover, in silico studies have shown that thymoquinone may have inhibitory activities against SARS-CoV-2 M^pro^ [47], which has been evaluated as one of the most attractive viral proteins and as a possible SARS-CoV-2 druggable target [22,48]. The remarkable toxicity of thymoquinone has encouraged the use of MH with artemisinins for the preparation of less toxic thymoquinone-based hybrids [17]. In particular, Tsogoeva’s research group reported extensive studies on the design and synthesis of artemisinin–thymoquinone hybrids successfully evaluated for anticancer, antiviral and antimalarial activities [49,50,51]. De Oliveira et al. [52] repurposed a selection of Tsogoeva’s artemisinin–thymoquinone hybrids as potential inhibitors of SARS-CoV-2 M^pro^ through a computational approach [53]. Among the hybrids considered in the referred study, two series of hybrids were depicted in Figure 5B: the series of dihydroartemisinin-derived hybrids **1** characterized by stable linkers of different lengths and the series of artesunate-derived hybrids **2** with cleavable ester linkers of different lengths. The safety profile of the hybrids was found to be improved in respect to the parent thymoquinone, which showed mutagenicity as well as carcinogenicity in both mouse and rat models. Indeed, all hybrids were negative for mutagenicity and hybrid **1** was also found to be negative for carcinogenicity in both mouse and rat models. Molecular docking studies showed a significant interaction between all hybrids considered and the active fraction of the enzyme of M^pro^ compared to some reference drugs, including remdesivir. The analyses of the physical–chemical properties of the hybrids considered indicated that the hybrids were able to permeate cell membranes wishing for a good pharmacokinetic profile. 

Hermann et al. reported the synthesis of a library of artesunate–quinoline hybrids obtained in a condensation reaction between artesunate and chloroquinoline derivatives (Figure 5C) [54]. Quinoline alkaloids are an important class of *N*-heterocyclic compounds showing many pharmaceutical properties, including antibacterial, antiviral, anticancer and antiparasitic effects [55,56]. Quinoline rings represent an attractive scaffold in rational drug design since they can improve physical and chemical properties as well as the pharmacological behavior of resulting hybrids. Among others, hybrid **3** and hybrids **4a–c**, which also contain a 1,2,3-triazole ring unit, were tested for cytotoxicity and antiviral activity against SARS-CoV-2 in African green monkey epithelial kidney cells Vero E6. All hybrids considered showed interesting inhibitory activity with EC_50_ in the range of 11–19 μM, outperforming unconjugated artesunate even though they were found to be ca. three to five-fold less active in respect to the reference drug remdesivir. On the other hand, hybrids **4a–c** were found to be significantly less cytotoxic in Vero E6 cells than both unconjugated artesunate and the reference drug remdesivir. 

Navacchia et al. repurposed some dihydroartemisinin–ursodeoxycholic bile acid hybrids (Figure 5D), already tested in selected cancer cell lines [57], for antiviral *anti*-SARS-CoV-2 activity evaluation [58]. Indeed, much evidence of the therapeutic potential of ursodeoxycholic bile acid in SARS-CoV-2 has been reported. Brevini et al. demonstrated in ex vivo experiments that ursodeoxycholic bile acid plays a role in downregulating ACE2 levels, thus reducing susceptibility to SARS-CoV-2 [59]. Ursodeoxycholic bile acid has also been reported to be beneficial in the regeneration of the damaged airway epithelium [60] and in the prevention of SARS-CoV-2 [61]. Hybrids **5** and **6** reported in Figure 5D were the most active of the series [57,58]. Hybrid **5** was prepared with click chemistry, leading to the formation of the stable triazole linker [57]. In turn, hybrid **6** was prepared with a direct condensation reaction between the dihydroartemisinin hemiacetal group and the ursodeoxycholic bile acid carboxylic moiety, leading to the formation of the corresponding fused hybrid through a cleavable ester bond [57]. Hybrids **5** and **6** were tested in Vero E6 as well as human epithelial lung cells Calu-3. Hybrid **5** was found to be the most effective compound in decreasing the SARS-CoV-2 load in a dose-dependent manner at all stages of viral infection with LR (log reduction) > 2.5 in Vero E6 and LR > 1.2 and 1.4 in Calu-3, respectively, in pre- and co-infection phases and LR = 4.97 in Vero E6 and LR = 4.04 in Calu-3 when administered post-infection. On the other hand, hybrid **6** showed the best effect on the pre-infection phase with LR = 1.84 in Vero E6 and LR = 1.50 in Calu-3. It is worth noting that both dihydroartemisinin and ursodeoxycholic bile acid alone exhibited LR ≤ 1 in all stages of the infection, thus demonstrating the effectiveness of MH. Moreover, hybrids **5** and **6** showed much lower cytotoxicity in both healthy Vero E6 and Calu-3 cells than the parent dihydroartemisinin. The in vitro study reported on both infected Vero E6 and Calu-3 cell lines revealed that click hybrid **5** can be considered a potential candidate for the post-infection treatment of SARS-CoV-2 infection due to a significant reduction of viral replication, no cytotoxicity and chemical stability. Preliminary mechanism studies indicated that the viral replication reduction showed by hybrid **5** after post-infection treatment may be ascribable to the down-regulation of ACE2 expression, possibly via the inhibition of Farnesoid X receptor signaling, as reported in the literature for ursodeoxycholic bile acid [59].

### 3.2. Peptidomimetic Inhibitor 2-Pyrrolidone-Based Hybrids

Several viral proteins as potential SARS-CoV-2 druggable targets have been evaluated. Among these, M^pro^ has been reported as the most appealing target for drug design being a highly conserved viral protein commune to all coronaviruses, not present in mammalian cells [62]. Indeed, the covalent inhibition strategy has been extensively applied for the development of small-molecule peptidomimetic hybrid M^pro^ inhibitors [63]. 

**Figure 6:**
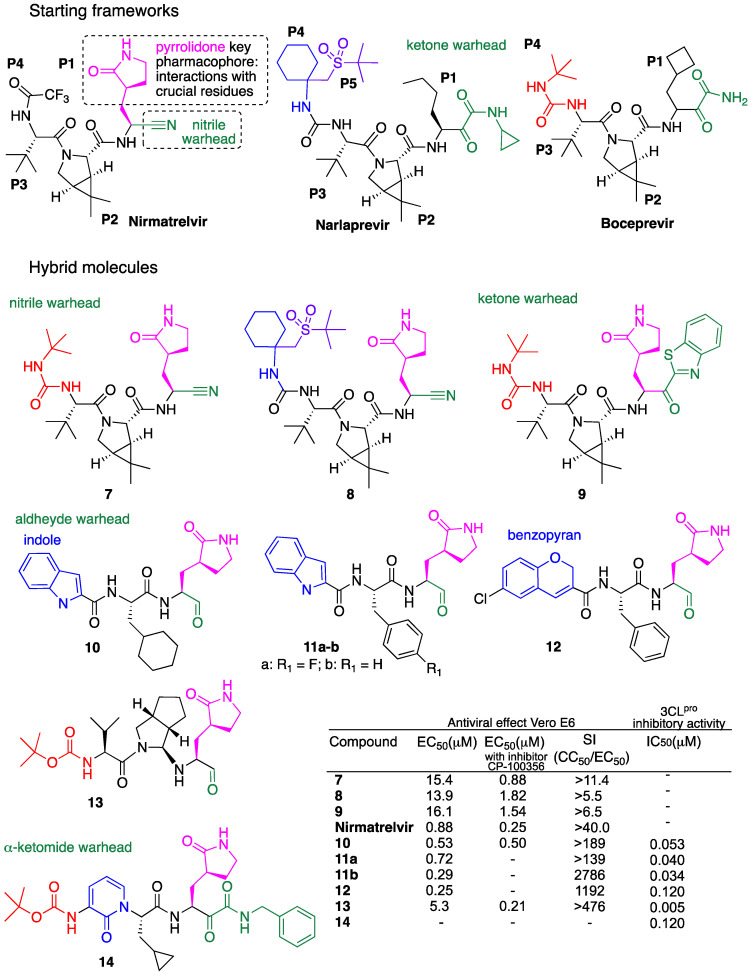
Peptidomimetic-based hybrids: molecular structures of starting frameworks; molecular structures of hybrids and selected biological data.

In particular, Pfizer discovered some potent peptidomimetic M^pro^ inhibitors of SARS-CoV-2, and among which, nirmatrelvir (Figure 6) was developed for oral administration and commercialized in combination with ritonavir as paxlovid [6,7,8]. This result has strongly addressed the research of antiviral *anti*-SARS-CoV-2 drugs towards the design of structure-based peptidomimetic hybrids targeting the SARS-CoV-2 M^pro^. Due to the extensive research and the related review papers already published on this topic [64,65,66], we reported herein only a representative selection of peptidomimetic hybrids characterized by the presence of the pyrrolidone key pharmacophore in P1 and different warhead groups such as nitriles, ketones, aldehydes and α-ketoamides (Figure 6).

An approach to peptidomimetic hybrid molecules effective against SARS-CoV-2 is represented by the rational design reported by Kneller et al. [67] of new hybrids based on hepatitis C virus protease inhibitors boceprevir or narlaprevir [68,69] and nirmatrelvir (Figure 6). Hybrids **7-9,** depicted in Figure 6, were obtained by shuffling pyrrolidone key pharmacophore, which is characteristic of the reference drug nirmatrelvir, to boceprevir and narlaprevir scaffolds and by changing the aldehyde warhead moiety to a nitrile or an electrophilic arylketone group. In particular, hybrids **7** and **8** present the peptidomimetic structure of boceprevir and narlaprevir, respectively, as well as the same pyrrolidone and nitrile warhead moieties of reference drug nirmatrelvir (Figure 6). In turn, hybrid **9** presents the peptidomimetic structure of boceprevir, the same pyrrolidone of hybrids **7** and **8** and the reference drug nirmatrelvir in position P1, but a different warhead such as an electrophilic arylketone (Figure 6). Thermodynamic measurements demonstrated that hybrids **7** and **8** effectively inhibit the M^pro^ in vitro. Antiviral activity studies in Vero E6 infected cells showed that all hybrids tested are up to 16-18-fold less active than the reference drug nirmatrelvir with EC_50_ in the range of 14-16 μM (Figure 6). The antiviral activity of hybrids **7**–**9** as well as of nirmatrelvir was also assessed in the presence of CP-100356 P-glycoprotein inhibitor. Under these conditions, the antiviral activity was found to be significantly improved in all cases. In particular, hybrid **7** showed a 17-fold increase in antiviral potency with EC_50_ in the same order of magnitude as the reference drug nirmatrelvir (EC_50_ = 0.88 and 0.25, respectively) (Figure 6). 

Dai et al. reported the design and synthesis of several peptidomimetic hybrids characterized by an aldehyde warhead with high inhibitory activity against M^pro^ [70,71]. The authors reported first the synthesis of hybrids **10** and **11a**, depicted in Figure 6 [70]. Both hybrids **10** and **11a** showed potent inhibitory activity against SARS-CoV-2 M^pro^ with EC_50_ = 0.53 and 0.72 μM, as well as excellent antiviral activity with IC_50_ = 0.053 and 0.040 μM, respectively. In vivo pharmacokinetic studies in mice allowed for the identification of **11a** as the lead compound. Further in vivo pharmacokinetic and toxicity studies of **11a** were performed in Sprague Dawley rats and beagle dogs to identify **11a** as good candidates for clinical studies. In a following paper, the same authors reported on the rational design and synthesis of a series of peptidomimetic hybrids having the same phenyl ring at P2 and an aldehyde warhead, but different pharmacophore units at P3 [71]. Hybrids **11b** and **12**, depicted in Figure 6, were the most active series as inhibitors of SARS-CoV-2 M^pro^, with IC_50_ = 0.034 μM and 0.120, respectively. Among all, hybrids **11b** and **12** also showed the best antiviral activity with IC_50_ = 0.29 and 0.25 μM, and selectivity indexes (SI) of 2786 and 1192, respectively. The preliminarily results in mice after intraperitoneal, subcutaneous and intravenous administration of hybrid **11b** indicated a good pharmacokinetics profile, and therefore, hybrid **11b** could also be considered an interesting starting point for further optimization studies. 

More recently, Summa et al. reported the design and synthesis of a series of peptidomimetic hybrids with an aldehyde warhead and a variously substituted proline moiety at P2 [72]. Overall, hybrid **13**, reported in Figure 6, was considered the most active one. In particular, hybrid **13** showed excellent SARS-CoV-2 M^pro^ inhibition in the low nM range with IC_50_ = 5.0 nM, good antiviral activity in Vero E6 infected cells with IC_50_ = 5.3 μM that decreased up to 0.21 μM in the presence of CP-100356 P-glycoprotein inhibitor and high SI (>476).

Hilgenfeld et al. reported the synthesis of a hybrid series characterized by an α-ketoamide warhead [73]. Among all, pure (S,S,S)-diastereomer hybrid **14** [73,74], reported in Figure 6, was the most active inhibitor of SARS-CoV-2 M^pro^, with IC_50_ = 0.12 μM [74]. The antiviral activity evaluated in Calu-3 infected cells was found to be dose-dependent, with EC_50_ = 2.4 μM. Hybrid **14** also showed interesting peroral as well as inhalation bioavailability [74]. 

### 3.3. 1,2,3-Triazole-Based Hybrids

The emerging field of click chemistry offers a unique approach to the synthesis of new hybrid molecules through the formation of the 1,2,3-triazole ring, stable to metabolic degradation. Although the 1,2,3-triazole ring is primarily considered a linker and a bioisoster since it mimics different functional groups, the triazole moiety is more than just a passive linker and its role as a pharmacophore has been recognized [75,76]. Indeed, the 1,2,3-triazole is capable of hydrogen bonding and dipole interactions, which can be favorable in the binding of biomolecular targets. Despite the 1,2,3-triazole moiety not occurring in nature, the synthetic molecules that contain the 1,2,3-triazole unit show diverse biological activities [77].

**Figure 7:**
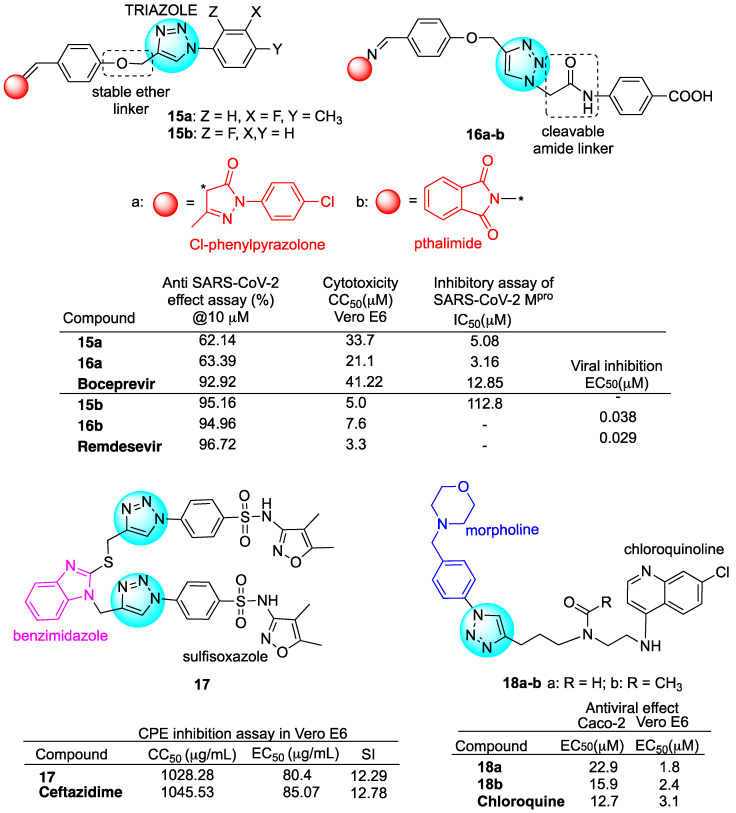
Molecular structures of 1,2,3-triazole-based hybrids and selected biological data.

Pyrazolone, a five-membered heterocycle with two adjacent nitrogen atoms (Figure 7), is a synthetic structural motif widely employed in medicinal chemistry for the development of new hybrid molecules with various biological activities due to remarkable therapeutic effects and robust pharmacological potency [78]. In particular, several molecules containing the pyrazolone ring have been reported to display antiviral activity against coronaviruses [79,80,81]. Aouad et al. reported a library of Cl-phenylpyrazolone–1,2,3-triazole hybrids, synthesized with a click reaction, that presents a stable ether linkage between the two pharmacophore units [82]. In turn, the 1,2,3-triazole moiety was further functionalized with lipophilic aryl-substituted groups by direct linkage or through an amide linker (**15a** and **16a**, Figure 7) [82]. All hybrids were investigated for cytotoxicity in Vero E6 cells as well as for antiviral and M^pro^ inhibitory activity against SARS-CoV-2 and compared with the reference drug boceprevir. The hybrids that present the amide linker were found to be more effective with respect to the non-amide ones. In particular, hybrid **15a** was found to be the most active of the non-amide series and hybrid **16a** was found to be the most active compound of the amide series (Figure 7). Hybrids **15a** and **16a** showed the same inhibition of replication (62% and 63%, respectively) but hybrid **16a** was more effective in M^pro^ inhibition, with IC_50_ of 3.16 μM, with respect to 5.08 μM reported for hybrid **15a** (Figure 7). Computational studies for the prediction of drug-likeness and ADME properties were also carried out for hybrid **16a**. Hybrid **16a** showed greater potential drug-like value; a similarly low risk for mutagenicity, teratogenicity and irritant effects; but a higher risk for reproductive effects compared to the reference drug boceprevir. In a following paper, the same authors reported the synthesis of a series of 1,2,3-triazole hybrids by replacing the Cl-phenylpyrazolone pharmacophore with a phthalimide moiety with the aim to improve the protein binding and the *anti*-SARS-CoV-2 activity [83] (**15b** and **16b**, Figure 7). Indeed, phthalimide has been employed in the design of potential anticancer, antimicrobial and *anti*-inflammatory drug candidates [84,85,86]. Similarly to the previous work, all hybrids were tested in Vero E6 cells. Among all, hybrids **15b** and **16b**, depicted in Figure 7, were the most active of the series, showing almost comparable antiviral activity to the reference drug remdesivir at 1 μM concentration (83.58, 87.82 and 92.72%, respectively). In particular, hybrid **16b** showed the highest viral inhibition among all tested compounds with EC_50_ = 0.038 μM, comparable to that of the reference drug remdesivir (EC_50_ = 0.029 μM) (Figure 7). Selected hybrids were also assayed in vitro for M^pro^ inhibition. Differently from the corresponding Cl-phenylpyrazolone-based hybrids, the tested hybrids with the phthalimide scaffold were found to be ineffective in M^pro^ inhibition (Figure 7). Lopinavir and boceprevir were used as reference drugs for ADMET studies on a selection of hybrids. Among others, hybrid **16b** showed a safe toxicity profile and a drug score of 0.28 compared to 0.17 for lopinavir and 0.37 for boceprevir.

Al-Humaidi et al. reported a study on benzimidazole–1,2,3-triazole hybrids containing a variety of pharmacophore units such as 1,2,4-triazole-3-thione derivatives, isatin and sulfisoxazole [87]. Among others, hybrid **17**, depicted in Figure 7, which contains the sulfisoxazole core characterized by antimicrobial activity, was the most promising one.

Ceftazidime, a promising potential *anti*-SARS-CoV-2 drug, was chosen by the authors as the reference drug [88]. Hybrid **17** exhibited the best binding score (−7.27 Kcal/mol) among all test compounds and a significantly higher binding score than the reference inhibitor ceftazidime against the SARS-CoV-2 spike protein. Hybrid **17** also showed strong and stable interactions with the spike Receptor Banding Domain (RBD) while exhibiting no binding interactions with ACE2 receptors. Similar results were obtained against the Omicron spike protein. Hybrid **17** was assayed in vitro for enzymes as well as cytopathic inhibition in human Vero E6 cells. The compound showed similar IC_50_ values against both the SARS-CoV-2 spike protein (74.51 nM) and the spike protein of the Omicron variant (75.98 nM). Moreover, the cytopathic (CPE) inhibition assay revealed that SARS-CoV-2 was effectively inhibited by hybrid **17** with a high selectivity index (SI) (Figure 7). Other than the antiviral activity, hybrid **17** was found to exert a significant *anti*-inflammatory activity by reducing the cytokines’ (IL-1 and IL-6) protein expression levels. The overall data proved the interesting activity of hybrid **17**.

Tsogoeva et al. reported the synthesis and inhibitory profile of novel quinoline–morpholine hybrids **18a**–**b** embedding a 1,2,3-triazole ring as a stable linker (Figure 7) [89]. The quinoline–morpholine hybrids **18a**–**b** tested in both Caco-2 and Vero E6 cells displayed similar or stronger *anti*-SARS-CoV-2 activity with respect to the reference drug chloroquine (Figure 7).

### 3.4. Thiazole Analogs and Coumarin-Based Hybrids

**Figure 8:**
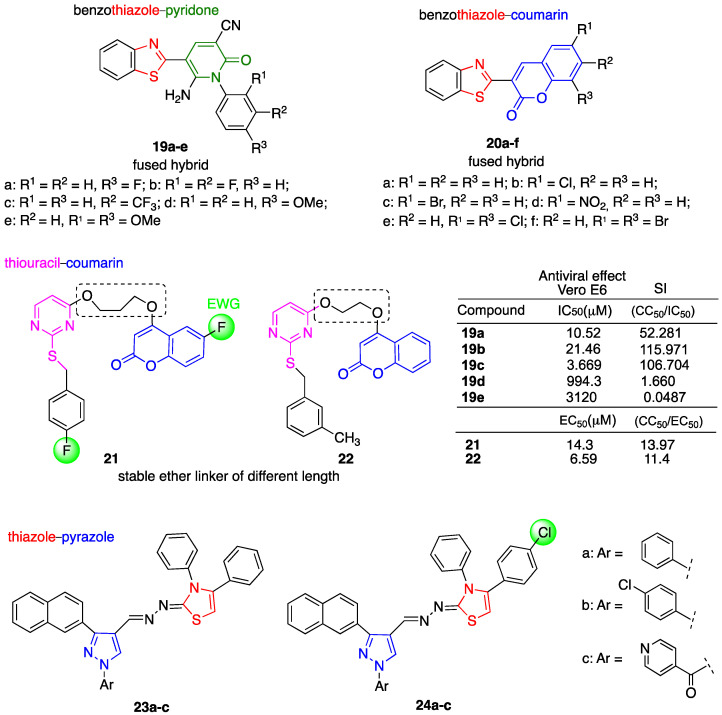
Chemical structure of benzothiazolyl–pyridine, benzothiazolyl–coumarin, thiouracil–coumarin, thiazole–pyrazole hybrids and selected biological data.

The synthesis and in vitro evaluation of the antiviral activity of a novel series of benzothiazolyl–pyridine [90] as well as of benzothiazolyl–coumarin [91] hybrids were reported (Figure 8). The rationale of these studies lies in the fact that the benzothiazoles have shown potential antiviral activity [92] and that the MH of two or more diverse heterocyclic moieties can lead to a new hybrid molecule with enhanced bioactivity [93].

Metwally et al. [94] reported the synthesis of 5-benzothiazolyl-1-(aryl)-pyridine-2-ones **19a-e** in which the phenyl ring was substituted with one or more methoxy electron-donating groups and one or more electron-withdrawing groups such as bromine, chlorine and fluorine atoms. This study demonstrated that the introduction of one halogen-withdrawing group at the 4-position of the benzene ring increased the *anti*-SARS-CoV-2 activity in Vero E6 infected cells (4-Cl: IC_50_ = 142.2 μM < 4-Br: IC_50_ = 73.84 μM < 4-F: IC_50_ = 10.520 μM) in respect to the introduction of a methoxy group (4-OMe: IC_50_ = 994.3 μM). On the other hand, the introduction of a second donating or withdrawing group at the 2-position dramatically decreased the antiviral activity (Figure 8). Among halogen atoms, the introduction of fluorine gave the highest activity. The best result was obtained by increasing the number of atoms of fluorine at the 3-position by introducing a trifluoromethyl group (hybrid **19c**, Figure 8). Further mechanism studies of *anti*-SARS-CoV-2 activity were performed on hybrids **19a–c**. In particular, hybrid **19a** was found to reduce the virucidal inhibition by ca. 50% and ca. 25% at 10 and 0.1 μM, respectively. On the one hand, hybrids **19b,c** were found to reduce the virucidal inhibition by ca. 80% independent of the concentration in the range of 10-0.1 μM. SARS-CoV-2 3CL^pro^ inhibition was also evaluated in the case of hybrids **19a–c** and compared with that of the reference drug lopinavir (IC_50_ = 129.8 μg/mL). Hybrid **19c** (3-CF_3_: IC_50_ = 240.6 μg/mL) showed higher SARS-CoV-2 3CL protease inhibition than **19a** (4-F: IC_50_ = 544.6 μg/mL) and **19b** (2,3-F: IC_50_ = 868.2 μg/mL). The overall data suggest that fluorine-containing hybrids **19a–c** could be considered lead compounds. In particular, the presence of the trifluoromethyl group can be relevant for the inhibition of viral replication in Vero E6 infected cells. It is worth noting that hybrids **19a–c** displayed low toxicity in the host cells, as evidenced by the SI values reported in Figure 8.

Abdallah et al. [91] reported the synthesis of a series of benzothiazolyl–coumarin hybrids. It is worth noting that coumarins are considered interesting combination partners for MH due to their potent biological activity [17]. Moreover, a recently reported molecular docking investigation showed that natural coumarin analogs displayed a remarkable inhibition ability against SARS-CoV-2’s main protease [95]. The authors reported the synthesis of a series of hybrids **20a–f** with different substituents, such as electron-withdrawing groups (NO_2_, Cl, Br, etc.) on the phenyl ring of the coumarin moiety (Figure 8). All hybrids have been fully characterized and molecular docking and binding energy investigations allowed for the demonstration of a promising binding with nucleocapsid protein NI63 (PDB ID: 5epw). In particular, hybrid **20f** was the most potent of the series as it forms five bonds. The presence of two bromine atoms was responsible for the enhanced activity.

In turn, Vishwanath et al. [96] reported the conjugation of coumarin with a thiouracil moiety in order to obtain *anti*-SARS-CoV-2 hybrids able to inhibit RdRp. The rational design of the thiouracil–coumarin hybrids reported was based on the ether linkage of different lengths, different numbers and the nature of substituents on the phenyl ring linked to the thiouracil unit and on the phenyl ring of coumarin moiety. Hybrids **21** and **22**, depicted in Figure 8, were the most active of the series with EC_50_ = 14.3 and 6.59 µM, respectively, in Vero E6 infected cells and no significant toxicity to host cells. All hybrids were also tested on some SARS-CoV-2 variants such as D614G and B.617.2. Some of the hybrids were found to be effective against both variants with IC_50_ < 10 μM; for instance, hybrid **21** showed IC_50_ < 10 μM with SI = 20 in both cases. The in silico molecular interaction studies evidenced that hybrid **22** has a common catalytic site of RdRp with the antiviral drug remdesivir but not with suramin, which is an inhibitor of SARS-CoV-2 RdRp. The in silico ADMET property studies of hybrids **21** and **22** showed that the maximum recommended therapeutic dose of both hybrids was comparable to that of the antiviral drug remdesivir. Some pharmacokinetic studies were also carried out for lead hybrid **21**. Male Wistar albino rats were treated by a single oral gavage administration at a dose of 10 mg/kg, which gave a plasma Cmax of 0.22 μg/mL and a final elimination half-life time of 73.30 h.

Seliem et al. reported the synthesis of a series of novel pyrazolothiazole hybrids that were evaluated for their potential inhibitory activity of SARS-CoV-2 M^pro^ following the virtual screening strategy [97]. Both combination partners pyrazole and thiazole as well as pyrazolothiazole derivatives have been reported to exhibit broad biological activity [98,99] and are pharmacophore units present in antiviral drugs such as pyrazofurin and ritonavir. In particular, the interactions of the hybrids with SARS-CoV-2 M^pro^ were evaluated through in silico molecular docking. The molecular docking of hybrids **23a–c** and **24a–c**, depicted in Figure 8, within the binding pocket of SARS-CoV-2 M^pro^ showed the high potency of ligands **23a–c** and **24a–c** due to their ability to form stable protein–ligand complexes strengthened by hydrogen bonding and interactions such as hydrophobic, Pi–alkyl, Pi–Pi stacking, Pi–sigma and Pi–sulfur. These findings indicate that hybrids **23a–c** and **24a–c** can be candidates for further in vitro studies.

### 3.5. Miscellanea

**Figure 9:**
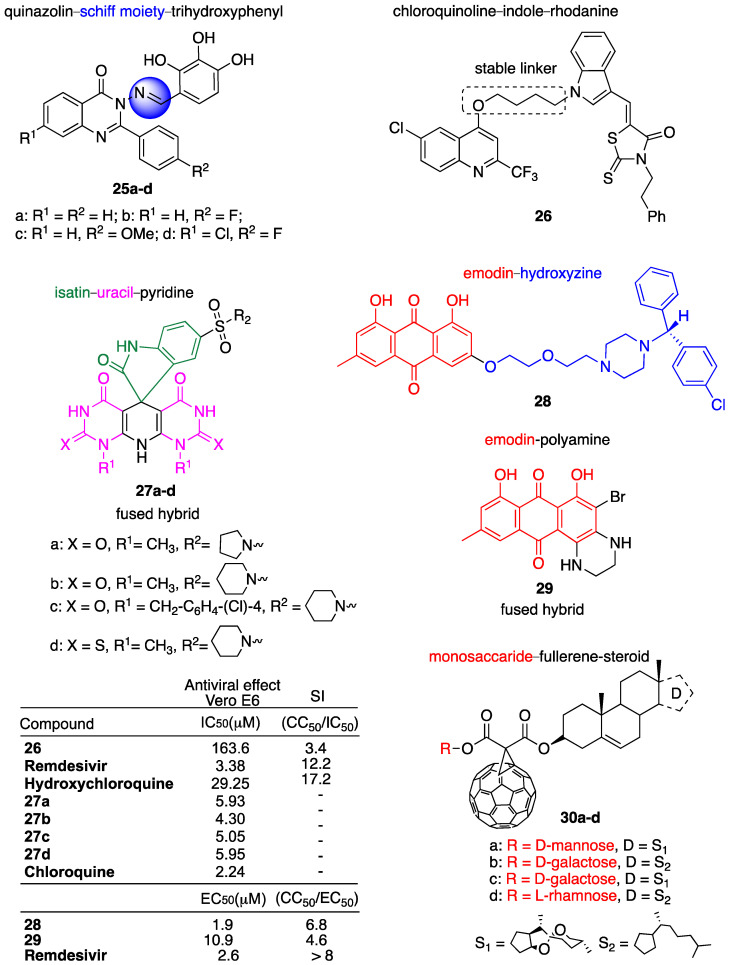
Chemical structure of hybrids and selected biological data.

Abdel-Rahman et al. repurposed a series of quinazoline–trihydroxyphenyl Schiff base hybrids, previously evaluated in vitro for the inhibition of phosphodiesterase and antiproliferative activity in some cancer cell lines [100], as potential inhibitors of M^pro^ SARS-CoV-2 and RdRp [101]. Quinazoline represents an important class of N-heterocyclic compounds with a wide range of pharmaceutical properties, including antiviral effects [56,102,103]. On the other hand, the ability of the trihydroxyphenyl moiety to act as a metal chelator can help bind the excess of intracellular iron present in the case of COVID-19 infection derived from iron dysmetabolism. Hybrids **25a–d**, depicted in Figure 9, were studied in silico. Molecular docking studies showed that both the quinazoline moiety and tri-hydroxy group may be effective in the inhibition of SARS-CoV-2 M^pro^ and RdRp. In particular, molecular docking showed the high potency of ligand hybrid **25b** characterized by the presence of a fluorine atom on the quinazoline side. Furthermore, the authors explored pharmacokinetic and toxicological properties via ADMET. All hybrids showed significant values for oral absorption. Among others, hybrid **25c** showed the best water solubility and no mutagenicity. However, all hybrids were found to be significantly toxic in T. pyriformis.

Panda et al. reported [104] the design and synthesis of a series of hybrids obtained by MH of quinoline [55], indole [105] and rhodanine [106], which are privileged scaffolds in medicinal chemistry. A trifluoromethyl group was also introduced in position 2 of the quinoline ring. It is worth noting that fluorine has been playing a relevant role in modern pharmaceuticals [107]. All hybrids were tested in Vero E6 normal and infected cells. Hybrid **26**, depicted in Figure 9, showed the best activity and SI even though it was significantly less active than the reference drugs hydroxychloroquine and remdesivir (data reported in Figure 9). ADME studies indicated that all hybrids had good water solubility and intestinal absorption as well as plasma protein binding.

Fayed et al. reported the synthesis of novel spiro-oxindoles based on the MH of isatin, pyridine and a pyrimidine scaffold such as uracil [108]. As reported by the authors, the combination partners chosen are on the basis of some antiviral drugs such as arbidol (isatin), chloroquine (pyridine) and acyclovir (uracil). All hybrids were tested for antiviral activity in Vero E6 cells using chloroquine as the reference drug. Hybrids **27a–d**, reported in Figure 9, were the most active of the series with IC_50_ in the range of 4.30–5.95 μM, and were thus 2–2.6 times less active than the reference drug chloroquine (IC_50_ = 2.24 μM) (Figure 9). Viral replication inhibition percentage was also assessed for all hybrids at different concentrations. In particular, hybrids **27a–d** showed 84, 99, 80 and 91% inhibition, respectively, at 5 μM concentration. Mechanisms of action such as inhibition of RdRp and spike glycoprotein were also investigated for hybrids **26a–d** using chloroquine as the reference drug. Hybrids **27a–d** exhibited potent inhibitory activity towards RdRp with IC_50_ in the range of 40.30–44.90 nM vs. IC_50_ = 45 nM for chloroquine, and towards spike glycoprotein with IC_50_ in the range of 40.27–44.83 nM vs. IC_50_ = 45 nM for chloroquine.

Meunier et al. reported the synthesis and biological evaluation of two series of hybrids based on an emodin scaffold covalently linked to diphenylmethylpiperazine derivatives such as norchloryclizine, hydroxyzine (Figure 9) and cetrizine, and to alkyl polyamines of different length [109]. Emodin is an anthraquinone-based natural product with a broad range of biological activities still in use by traditional Chinese medicine. Other than the wide spectrum of pharmacological effects, for instance, anticancer, antiviral and antibacterial, emodin shows poor bioavailability and significant toxicity that limit a possible clinical use [110]. MH can help to overcome emodin’s drawbacks as well as to exploit its therapeutic potential. Diphenylmethylpiperazine derivatives are *anti*-histamine drugs, whereas alkyl polyamines were chosen as combination partners due to their regulatory roles in immune cell functions, and therefore, to counteract the cytokine response. Hybrids **28** and **29**, reported in Figure 9, were the most active of each series. All hybrids were tested in Vero E6 infected cells. Among all, hybrid **28** showed the best antiviral *anti*-SARS-CoV-2 activity with EC_50_ = 1.9 μM and SI = 6.8 comparable to that of the reference drug remdesivir (EC50 = 2.6 μM, SI > 8). Moreover, hybrid **28** exhibited inhibition of viral replication > 90% at 6.25 μM.

Molecular docking and dynamics simulation has revealed the potential inhibitory activity of fullerene C60 against SARS-CoV-2 by blocking the target protein 3CLpro, M^pro^ and RdRp [111]. In this light, Suarez et al. [112] repurposed the C60 scaffold for MH with steroids and monosaccharides in order to improve C60 biological activity and bioavailability. Hybrids **30a–d**, depicted in Figure 9, were synthetized and fully characterized. The molecular docking studies suggested that hybrids **30a–d** are able to inhibit the M^pro^, and therefore, the possible application of these compounds as *anti*-SARS-CoV-2 might be considered.

**Figure 10:**
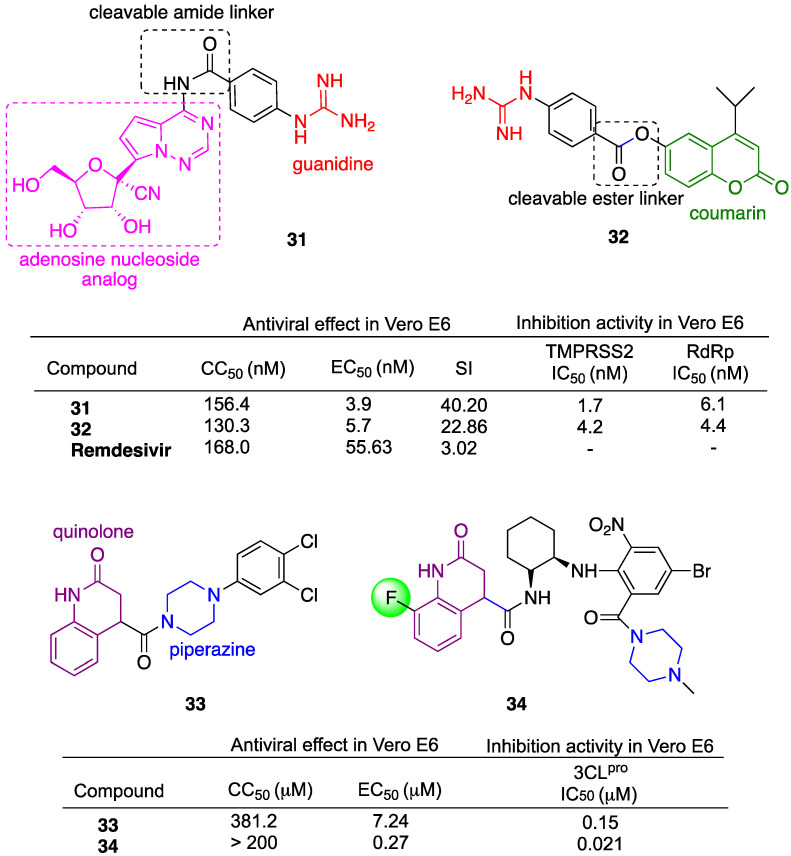
Chemical structure of hybrids and selected biological data.

Hamdy et al. reported the design and synthesis of a library of new hybrids with the aim of obtaining multiple targeting molecules [113]. The hybrids were designed to target SARS-CoV-2 RdRp, required for viral replication, as well as human transmembrane serine protease TMPRSS2, required for spike protein activation and viral entry. The dual inhibitor approach can also help to overcome future drug resistance. The rational design is based on the use of a guanidine moiety that mimics the natural substrate L-arginine at the cleavage site of the protein. Moreover, phenyl-guanidine derivatives have displayed therapeutic potential [114]. The phenyl-guanidine scaffold was conjugated with nucleoside/nucleobase analogs through an amide cleavable bond or coumarin-like moiety through a cleavable ester bond. Nucleosides as well as nucleoside analogs can behave as antimetabolites and can inhibit cellular division and viral replication by their incorporation into DNA or RNA, resulting in potential antiviral agents [115]. Hybrid **31**, incorporating the same adenosine analog present in remdesivir, and hybrid **32**, containing coumarin pharmacophore, were the most active of the series (Figure 10). All hybrids were tested in Vero E6 cells. In particular, hybrids **31** and **32** showed significantly higher antiviral activity compared to remdesivir, with IC_50_ 12-16-fold lower as well as an improved SI (Figure 10). Moreover, hybrids **31** and **32** displayed significant inhibition activity with IC_50_ in the low nM range against both TMPRSS2 and RdRp (Figure 10).

Zhou et al. reported the design and synthesis of novel hybrid 3CL^pro^ inhibitors [116,117]. In their first paper, the authors reported the discovery of hybrid **33** (Figure 10) after high-throughput screening of SARS-CoV-2 3CL^pro^ [116]. Hybrid **33** presents a 2-quinolone unit belonging to a versatile therapeutic compound class [118] and a piperazine moiety. Hybrid **33** showed a high inhibitory effect against 3CL^pro^, good *anti*-SARS-CoV-2 activity and negligible cytotoxicity in Vero E6 cells infected by the SARS-CoV-2 Delta variant (Figure 10). Hybrid **33** was also tested in mice for pharmacokinetic evaluation with good results. In vivo assays in a K18-hACE2 mice model infected by the SARS-CoV-2 Delta variant confirmed good 3CL^pro^ inhibitory and antiviral activities at a dosage of 300 mg/kg twice a day by intraperitoneal injection. In their second paper, the authors reported the rational design of a library of hybrids based on deep insight into the structure–activity relationship of hybrid **33** [117]. The hybrids were tested in Vero E6 cells infected by the SARS-CoV-2 Delta variant. Among all, hybrid **34** embedding a 2-quinolone fluorine-derivative was the most active (Figure 10). Hybrid **34** showed antiviral activity and 3CL^pro^ inhibition ca. 26 and 7 times higher than hybrid **33**, respectively. Hybrid **34** efficacy was also investigated in vivo in a K18-hACE2 mice model infected by the SARS-CoV-2 Delta variant. Hybrid **34** was tested for toxicity in vivo upon the oral administration of 500 mg/kg twice a day, displaying a safe toxicity profile. Oral treatment with a hybrid **34** compound at a dose of 200 mg/kg significantly reduced lung viral copies in a K18-hACE2 transgenic mouse model. The overall data both in vitro and in vivo indicated hybrid **34** as the best lead compound in respect to the previously reported hybrid **33**.

## 4. Conclusions

Researchers have made a great effort to discover new antiviral *anti*-SARS-CoV-2 drugs by either isolation from natural resources or the synthesis of organic small molecules over the past five years. Drug repurposing strategies have also been considered as a viable alternative to the more usual and time-consuming drug development process. This present review is limited to small hybrid molecules obtained by the conjugation of both natural and synthetic compounds that exert *anti*-SARS-CoV-2 activities. Based on the data reported, a selection of hybrids could be considered lead compounds for the development of new drugs for treating COVID-19 infection, although a lack of in vivo assays should be underlined. Among all, the main protease inhibitors emerged as the most appealing target drugs. In particular, peptidomimetic hybrids have been receiving much attention due to the successful marketing of nirmatrelvir, the first oral drug to treat severe forms of COVID-19. Some of the reviewed papers evidenced the relevance of computational chemistry in the identification of scaffolds able to lead to new potential drugs.

COVID-19 therapeutics remain an active area of research due to the persistence of COVID-19 infection in the world and the continuous rise of new variants even though the mitigation of the virus’ effects due to booster vaccinations has strongly limited the clinical development of lead compounds. Meanwhile, information on the clarification of structure–activity relationships together with the rational application of new technologies and strategies such as artificial intelligence can contribute to identifying novel antiviral drugs that meet the requirements of the clinical treatment of COVID-19 as well as other virus-related diseases in order to prevent the future development of pandemics.
